# Common femoral vein wall thickness as a supportive imaging marker in the differential diagnosis of Behçet’s uveitis

**DOI:** 10.55730/1300-0144.6209

**Published:** 2026-02-12

**Authors:** Seda KUTLUĞ AĞAÇKIRAN, Esra KARDEŞ, Abdulbaki AĞAÇKIRAN, Haner DİRESKENELİ, Fatma ALİBAZ ÖNER

**Affiliations:** 1 Division of Rheumatology, Department of Internal Medicine, Faculty of Medicine, Marmara University, İstanbul, Turkiye; 2 Department of Ophthalmology, St. Johannes Hospital, Dortmund, Germany; 3 Department of Radiology, Faculty of Medicine, Bahçeşehir University, İstanbul, Turkiye

**Keywords:** Behcet’s disease, uveitis, diagnostic imaging, ultrasonography, differential diagnosis

## Abstract

**Background/aim:**

Ocular involvement is a major cause of morbidity in Behçet’s disease (BD), and its diagnosis may be challenging, particularly for patients with isolated ocular manifestations or incomplete fulfillment of diagnostic criteria. Increased common femoral vein (CFV) wall thickness measured by Doppler ultrasonography (US) has previously been shown to be a sensitive and specific marker for systemic BD. This study aimed to evaluate whether CFV wall thickness measurement may serve as a supportive tool in distinguishing Behçet’s uveitis (BU) from other forms of inflammatory uveitis.

**Materials and methods:**

This retrospective study included 45 patients with BU fulfilling the International Study Group criteria for BD and 72 patients with non-BD uveitis of infectious and noninfectious etiologies. Bilateral CFV wall thickness was measured using color Doppler US by a radiologist blinded to the clinical data. Group comparisons were performed and multivariate linear regression analyses were used to assess whether BU was independently associated with increased CFV wall thickness after adjustment for potential confounders. Exploratory analyses additionally evaluated the effects of disease duration and systemic treatment status.

**Results:**

Mean CFV wall thickness was significantly higher in the BU group than in the non-BU group in both extremities (p < 0.001). BU remained an independent predictor of increased CFV wall thickness after adjustment for sex and venous Doppler findings. Additional exploratory analyses adjusting for disease duration and current systemic treatment status did not change this association.

**Conclusion:**

CFV wall thickness measured by Doppler US was significantly increased in patients with BU compared to other forms of inflammatory uveitis. These findings suggest that CFV wall thickness may serve as a supportive or adjunctive imaging marker in selected cases to assist in the differential diagnosis of ocular BD.

## 1. Introduction

Behçet’s disease (BD) is a variable vessel vasculitis that affects the mucocutaneous, vascular, ocular, neurological, and gastrointestinal systems [1]. Ocular inflammation occurs in approximately two-thirds of BD patients, and sight-threatening ocular complications constitute the primary cause of disability in BD. The affected population is predominantly young and male, particularly within the first 2 years of disease onset [2–4]. Patients with BD typically develop recurrent nongranulomatous uveitis accompanied by obliterative retinal vasculitis, which can affect either the anterior or posterior segment of the eye, or both [4].

In the case of ocular BD involvement, multimodal imaging is crucial for the diagnosis of the disease. For assessments of the degree and severity of posterior segment involvement, Cunningham et al. emphasized the significance of routine color fundus photography and fluorescein angiography (FA) [2]. In the case of occlusive and leaky retinal vasculitis in ocular BD involvement, FA is still regarded as the gold standard for diagnosis and follow-up [5]. According to the established diagnostic criteria for BD, a diagnosis can be made by combining specific clinical findings and organ system involvement; however, it is evident that there are some limitations to the multisystemic criteria sets currently in use [6]. The diagnosis may be delayed in clinical settings because of the variable evolution of disease manifestations. The mean interval between initial manifestation and fulfillment of the diagnostic criteria may vary between 4 and 8 years [7,8]. It is also known that ocular involvement may be the initial manifestation in 10%–20% of BD patients before the diagnostic criteria are met [3]. The diagnostic process can be very challenging, particularly in patients who only have a single major organ involvement and do not fulfill diagnostic criteria.

Ocular involvement is a crucial part of the current diagnostic criteria, but the main challenge faced by many ophthalmologists is to rule out other infectious and noninfectious pathologies because of the nonspecific nature of ocular BD findings.

We recently demonstrated that increased common femoral vein (CFV) wall thickness measured by color Doppler ultrasound (US) is a distinctive feature of BD, rarely present in other inflammatory or vascular diseases. This variable has high sensitivity and specificity (above 80%) for the cut-off value of ≥0.5 mm, and we accordingly suggested the measurement of CFV thickness as an easy and noninvasive diagnostic test for BD [1,9,10]. However, the potential role of CFV wall thickness in the diagnostic evaluation of ocular BD, particularly for patients presenting with isolated uveitis, remains less clearly defined.

Despite the availability of various criteria sets and the presence of experienced ophthalmologists, there is an unmet need for tools to aid in early diagnosis and to minimize the risk of misdiagnosis in cases of ocular BD involvement. In this context, supportive diagnostic approaches that may assist clinicians during the differential diagnosis of ocular BD are of particular interest. For this reason, in this study, we aimed to evaluate the potential role of CFV wall thickness measured by color Doppler US as a supportive imaging marker in the differential diagnosis of ocular BD from cases of other forms of uveitis.

## 2. Materials and methods

Forty-five patients with Behçet’s uveitis (BU), meeting the criteria of the International Study Group for Behçet’s Disease (ISG) [11], and 72 patients who were followed with a diagnosis of uveitis caused by infectious and inflammatory etiologies other than BD (i.e., non-BU) in a tertiary ophthalmology clinic were included in this study. The study was based on retrospective, cross-sectional, single-center analysis. All patients were diagnosed and monitored by an expert ophthalmologist (E.K.). For all patients, data on their demographics, disease characteristics, and the type and duration of immunosuppressive treatment were collected from their medical charts retrospectively.

An experienced radiologist (A.A.), with more than 10 years of experience in vascular US and blinded to the clinical data and group allocation, performed bilateral lower extremity venous Doppler US on the same day as the routine ophthalmological visits. A high-resolution ultrasound Doppler system (SSA-790A, Toshiba Medical Systems Corporation, Otawara, Japan) equipped with a high-resolution linear transducer (8–12 MHz) was used for the measurement of CFV wall thickness in the craniocaudal direction. CFV wall thickness was measured in B-mode from the posterior wall of the CFV at 2 cm distal from the saphenofemoral junction, with the patient in a supine position. Both measurements were obtained during the same examination session using the same standardized technique. During these measurements, additional US findings including collateral development, chronic thrombotic changes, the presence of reflux, and recanalization were also noted. The average of two measurements was recorded as the CFV wall thickness. Based on previous studies demonstrating high diagnostic accuracy for BD, measurement values above 0.5 mm were accepted as diagnostic for BD [9]. In the context of the present study, this threshold was used to support the evaluation of ocular BD.

For intraobserver reliability, repeated measurements were performed on the same patients on the following day using the same measurement protocol, and the agreement was found to be good (intraclass coefficient for right CFV: 0.941 [95% confidence interval (CI): 0.761–0.985], p < 0.000; intraclass coefficient for left CFV: 0.939 [95% CI: 0.755–0.985], p < 0.000). Interobserver reliability was not formally assessed in this study; however, previous studies by our group using the same protocol reported good interobserver agreement, supporting the reproducibility of the CFV wall thickness measurement technique [9,10].

The study protocol was approved by the Marmara University Local Ethics Committee (approval date: 2 October 2020; protocol number: 09.2020.1082), and written informed consent was obtained from each patient. The study was performed according to the Declaration of Helsinki.

### 2.1. Statistics

Data were analyzed using IBM SPSS Statistics 26.0 (IBM Corp., Armonk, NY, USA). Categorical variables were summarized as frequencies and percentages and compared using the chi-square test or Fisher exact test as appropriate. Continuous variables were summarized using appropriate descriptive statistics, and between-group estimates were reported with corresponding 95% CIs.

Between-group comparisons of continuous variables were performed using the independent-samples t-test or one-way analysis of variance (ANOVA) when parametric assumptions were met. Distributional properties were assessed using the Kolmogorov–Smirnov test.

To evaluate whether BU independently predicted increased CFV wall thickness, multivariate linear regression models were constructed. The CFV wall thickness for each lower extremity was entered as the dependent variable. The primary regression model included disease group (BU vs. non-BU) and sex as independent variables, given the imbalance in sex distribution between groups.

Because individual venous Doppler abnormalities (chronic thrombosis, venous reflux or insufficiency, collateral formation, and recanalization) were observed in relatively small numbers of patients, these variables were combined into a single composite indicator reflecting the presence or absence of venous pathology. This composite variable was incorporated into extended regression models to assess its potential confounding effect.

Exploratory extensions of the multivariable models additionally included disease duration and current systemic treatment status, treatment duration, and treatment category (conventional immunosuppressive agents vs. anti-TNF therapy) to evaluate the robustness of the primary association and to assess potential residual confounding. These analyses were conducted for sensitivity purposes and were not intended as primary hypothesis-testing models.

Regression coefficients (β) with corresponding 95% CIs were reported to quantify adjusted effect sizes. Additionally, 95% CIs for between-group mean differences were calculated. The intraclass coefficient was used to determine intraobserver reliability. All statistical tests were two-sided and values of p < 0.05 were considered statistically significant.

## 3. Results

### 3.1. Demographic characteristics

Patient demographics are shown in Table 1. The mean age was similar in the BU and non-BU groups (38.1 ± 10.3 years vs. 41.7 ± 12.6 years, p = 0.09). There was a female predominance in the non-BU group (66.7%, n = 48) compared to the BU group (48.9%, n = 22) (p = 0.06). Among the patients of the BU group, all had oral aphthae, 32 (71%) presented with genital ulcers, 18 (40%) had acneiform lesions, and 13 (28.9%) had erythema nodosum. None of the BU patients had clinically overt major organ involvement other than ocular disease at the time of evaluation; however, asymptomatic chronic venous thrombotic changes were incidentally detected in 19 patients (42.2%) during US assessment, and none of these patients had received anticoagulant therapy.

### 3.2. Presenting symptoms of uveitis

As shown in Table 1, decreased visual acuity was the most common presenting symptom in both groups. The BU and non-BU groups demonstrated comparable disease durations and numbers of uveitis attacks, and unilateral involvement was common in both groups.

### 3.3. Current and previous treatments

Table 1 summarizes the current and previous treatment data of the patients. Twenty-five (55.6%) BU patients were treated with conventional immunosuppressive agents, the most common of which was azathioprine (24 patients, 53.3%). Of 25 patients (55.6%) who received anti-TNFs, all received adalimumab. Among the non-BU patients, conventional immunosuppressive agents were administered to 29 (40.3%), the most common of which was azathioprine (21 patients, 29.2%). Twenty-four (33.3%) of the non-BU patients received anti-TNFs. Among the non-BU patients receiving anti-TNFs, adalimumab was again the most frequently used agent.

### 3.4. Anatomic and etiological classification of uveitis

As presented in Table 1, the majority of the patients had panuveitis (70/117; 59.8%), followed by anterior uveitis (32/117; 27.4%) and intermediate uveitis (10/117; 8.5%). In addition, all BU patients had panuveitis. Among the noninfectious cases, BD was the leading diagnosis. Twenty-six patients (26/117, 22.2%) had idiopathic uveitis, 15 (15/117, 12.8%) had sarcoidosis, 10 (10/117, 8.5%) had ankylosing spondylitis, and 9 (9/117, 7.7%) had HLA-B27-positive uveitis without any systemic involvement. One patient (1/117, 0.9%) was diagnosed with herpetic uveitis. The other etiologies of uveitis (11/117, 9.4%) included 2 cases of rheumatoid arthritis (1 intermediate, 1 anterior uveitis), 2 cases of Vogt–Koyanagi–Harada disease, 1 case of idiopathic multifocal choroiditis, 2 cases of anterior uveitis of Crohn’s disease, 3 cases of Fuchs’ uveitis, and 1 case of anterior uveitis of systemic lupus erythematosus.

As shown in Figure, the mean CFV wall thickness was significantly greater in the BU group for both extremities. For the right CFV, the BU group demonstrated a mean thickness of 0.75 mm (95% CI: 0.72–0.77), whereas the non-BU group had a mean thickness of 0.46 mm (95% CI: 0.44–0.48) (p < 0.001). Similarly, the left CFV measurements were 0.74 mm (95% CI: 0.72–0.76) in the BU group and 0.46 mm (95% CI: 0.44–0.48) in the non-BU group (p < 0.001).

Due to the disparity in sex distribution between BU and non-BU patients, multivariate linear regression analysis was conducted to determine if the correlation between BU and increased CFV wall thickness persisted after adjusting for sex. BU continued to be an independent predictor of greater CFV thickness in both extremities, with β = 0.38 mm for the right CFV (p < 0.001) and β = 0.36 mm for the left CFV (p < 0.001). Male sex showed only a mild effect (right CFV: β ≈ 0.09 mm, p = 0.12; left CFV: β ≈ 0.08 mm, p = 0.15), indicating that the observed CFV thickening could not be explained by sex differences. The adjusted models demonstrated good explanatory power (R^2^ ≈ 0.60).

When venous Doppler abnormalities including chronic thrombosis, venous reflux/insufficiency, collateral formation, and recanalization were combined into a single composite variable indicating the presence or absence of venous pathology and added to the regression models, BU remained a strong independent predictor of increased CFV wall thickness. The presence of venous pathology demonstrated only a modest effect on CFV measurements and did not change the strength or statistical significance of the association between BU and CFV wall thickness.

Additional exploratory analyses adjusting for age, disease duration, and current systemic treatment characteristics similarly did not significantly alter the observed association between BU and CFV wall thickness. We observed that the CFV measurements of 10 patients from the non-BU group were above the cut-off value of 0.5 mm. Among these patients, 3 had idiopathic intermediate uveitis and 7 had idiopathic panuveitis. We reassessed these patients for other clinical manifestations of BD and 8 presented with oral aphthae while 1 had papulopustular lesions, but none had genital ulcers or erythema nodosum. Additionally, chronic venous thrombosis without recanalization was observed in 3 of these patients, and 2 of 3 had venous collaterals and signs of venous insufficiency. However, 4 patients without venous thrombosis also had venous insufficiency. After a detailed rheumatological examination, 5 patients fulfilled at least one of the diagnostic criteria sets, with fulfillment of only the International Criteria for Behçet’s Disease (ICBD) for 4 patients and fulfillment of both the ICBD and ISG criteria for 1 patient. Although 3 patients did not fulfill one of the two diagnostic criteria sets, they were diagnosed with BD by expert opinion. These reclassifications were evaluated separately and were not incorporated into the primary comparative analyses. Additional data are provided in Table 2.

## 4. Discussion

BU is a significant contributor to disease-related morbidity and disability during the course of BD. Therefore, diagnosis and differentiation of the disease from its mimickers have vital importance together with urgent immunosuppressive treatment. This study has primarily focused on exploring whether CFV wall thickness measured by Doppler US, a previously described highly sensitive and specific diagnostic method for BD, can be used as an adjunctive or supportive method in the differential diagnosis of ocular BD in daily practice. In this study, we showed that the CFV wall thickness of BU patients was greater compared to patients with other forms of inflammatory uveitis.

BU can be diagnosed by the combination of ocular and other systemic manifestations. However, diagnosis may be challenging in cases of single major organ involvement or in the presence of nonspecific findings such as oral aphthae and acneiform lesions, which are particularly prevalent in adolescents and young adults, the main age group for BD. According to various diagnostic criteria sets that have been suggested recently, for the diagnosis of BD, patients need to demonstrate a mixture of clinical manifestations in more than one organ system [6]. However, the diagnostic performance of these criteria was criticized by many experts because they include highly prevalent but not specific manifestations such as vascular and neurological involvements.

In all criteria sets, patients are scored according to ocular involvement; however, the definitions of the eye lesions are still vague and nonspecific [12]. In 2022, the SUN Working Group Classification Criteria for BU expanded the definition of “compatible uveitic syndrome” and added some exclusion criteria that are incompatible with the diagnosis of BD, but the requirement of fulfillment of the ISG criteria for the classification of BU remains one of the shortcomings of this criteria set [13]. These criteria sets should also be applied cautiously in clinical settings given the risk of overdiagnosis. According to the ICBD criteria, ocular involvement is assigned 2 points; therefore, the addition of common findings such as oral aphthae may lead to rapid fulfillment of diagnostic thresholds despite the limited specificity of these manifestations.

Significant numbers of uveitis experts believe that the diagnosis of ocular BD can be made without other organ manifestations [14]. Considering that the emergence of other BD-specific manifestations may take years in patients with only ocular findings or that the disease may remain limited to ocular involvement throughout a patient’s life, several attempts have been made to create diagnostic algorithms based solely on the combination of specific ocular findings with high discriminative power [15]. However, with these methods, which rely on ocular findings, only half of the cases can be identified with high diagnostic probability. Additionally, despite advances in ocular imaging that facilitate early diagnosis, monitoring, and follow-up [16], the expertise required for the interpretation of the results may not be generally available, especially in regions with low BD prevalence.

In this context, the use of additional diagnostic methods that may assist and facilitate clinical decision-making seems necessary. Consequently, CFV wall thickness measurement, which has been validated as a diagnostic marker for BD across multiple centers in Türkiye [17,18], may provide complementary information to help resolve challenges in clinical settings. This method is also helpful for the diagnosis of incomplete BD [19] and the differentiation of gastrointestinal involvement of BD from Crohn’s disease [20]. In addition, Doppler US, which is an accessible, easily applicable, and radiation-free imaging technique, allows measurements to be performed without any specific preparation or modification.

One of the most significant concerns regarding the differential diagnosis of BU is the risk of misclassifying other inflammatory uveitides, especially infectious uveitides, which can have catastrophic consequences with improper immunosuppressive treatment. These concerns are in accord with previous studies demonstrating substantial interobserver variability among uveitis experts when classifying ocular images as BD-related or attributable to other etiologies, with the diagnostic agreement ranging from 56% to 81% [12]. The majority of reviewers accurately identified particular ocular signs as indicating BD, including smooth-layered hypopyon, superficial retinal infiltration with retinal hemorrhages, and branch retinal vein occlusion with vitreous haze. However, numerous ocular pathologies caused by infectious uveitis have been misinterpreted as BD. For this reason, we recruited a heterogeneous non-BD group comprising cases of sarcoidosis, HLA-B27-associated or ankylosing spondylitis-associated uveitis, infectious uveitis, and idiopathic uveitis, reflecting the spectrum of real-life diagnostic challenges. Although most patients in the non-BU group had CFV thickness measurements below the cut-off of 0.5 mm, 10 patients exceeded this threshold. These patients were reevaluated in a hypothesis-generating manner and were not incorporated into the primary comparative analyses, thereby avoiding incorporation bias. From an ophthalmological perspective, these patients were initially classified as having idiopathic uveitis because their ocular presentations lacked features considered highly characteristic for BU and no systemic diagnosis could be established at the time of evaluation. Moreover, in real-life practice, patients presenting with isolated ocular symptoms may not yet have undergone comprehensive rheumatological assessment in the early stage of disease and systemic manifestations may still be evolving. This may lead to initial diagnostic uncertainty and reflects the challenges of diagnosing inflammatory uveitis at an early stage, particularly in settings where clinical experience with this rare condition may vary. Interestingly, 3 of these patients had chronic asymptomatic thrombotic changes; however, systematic etiological workups for thrombosis were not available at the initial assessment. This finding also supports evidence from previous research that asymptomatic venous thrombosis occurs in up to 6% of patients with BD [21]. Another striking finding was that signs of venous insufficiency were observed in the patients without venous thrombotic changes. This also accords with our earlier observations, which showed that BD patients without overt thrombosis have a high prevalence of venous insufficiency, probably as a sign of subclinical inflammation [22].

There are a few limitations of our study. First, pathergy testing could not be routinely performed for the majority of non-BU patients due to the retrospective study design; therefore, it was not included in the final diagnostic scoring. Second, we were unable to recruit patients who had posterior uveitis, which is included in the definitions of ocular lesions in all BD diagnostic and classification criteria sets, and there was only one patient with infectious uveitis; this may restrict the generalizability of our findings. Finally, Doppler US is operator-dependent, although all measurements in this study were performed by a single experienced radiologist, ensuring internal consistency. Although interobserver agreement was not assessed in the present study, prior validation of the same CFV measurement technique demonstrated good interobserver reliability, suggesting that this method can be reproducibly applied by experienced practitioners. Despite these limitations, the observed association between BU and increased CFV wall thickness remained robust after adjustment for multiple potential confounders, supporting the role of CFV wall thickness as a complementary imaging method.

In conclusion, despite advances in ocular imaging and expert clinical evaluation, the diagnosis of BD remains challenging due to its heterogeneous presentation and the variable disease course. Our findings suggest that CFV wall thickness measurement may serve as a supportive or adjunctive tool to assist clinicians in the differential diagnosis of ocular BD, particularly in complex cases where classical diagnostic criteria are not yet fulfilled.

## Figures and Tables

**Figure:**
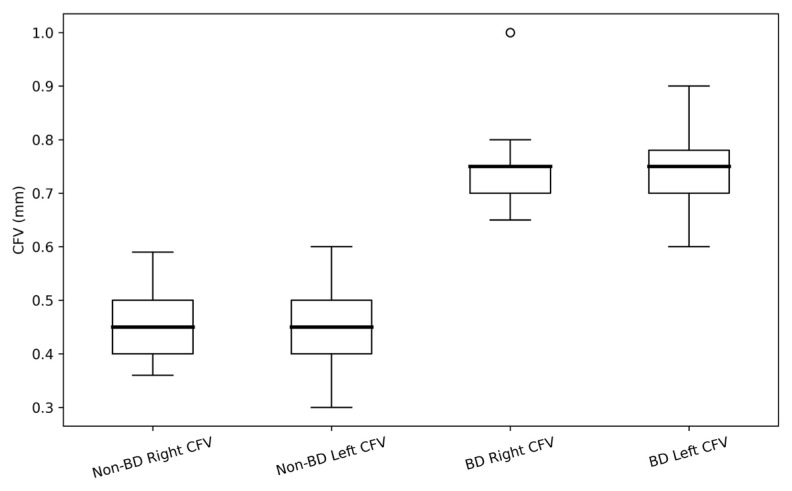
Comparison of common femoral vein (CFV) wall thickness of the patients with Behçet’s uveitis (BD) and non-Behçet’s uveitis (non-BD) for both legs. p < 0.001 between patients with Behçet’s uveitis and non-Behçet’s uveitis.

**Table 1:** Baseline demographics and disease characteristics of patients with Behçet’s uveitis and non-Behçet’s uveitis.

	Behçet’s uveitis (n =45)	Non-Behçet uveitis (n =72)
**Age (years), mean (SD)**	38.1 (10.3)	41.7 (12.6)
**Sex, n (%)**		
Male	23 (51.1)	24 (33.3)
Female	22 (48.9)	48 (66.7)
**BMI, kg/m** **^2^** **, mean (SD)**	24.9 (3.4)	28.3 (5.4)
**Duration of disease (years), mean (SD)**	8.8 (5.9)	6.3 (4.5)
**The number of uveitis attacks, median (min-max)**	3 (1–10)	2 (1–6)
**Laterality of uveitis, n (%)**		
Unilateral	30 (69.8)	52 (75.4)
Bilateral	13 (30.2)	17 (24.6)
**Ocular symptoms at presentation**		
Decreased visual acuity, n (%)	28 (87.5)	45 (78.9)
Hyperemia, n (%)	9 (28.1)	20 (35.1)
Painful vision, n (%)	5 (15.6)	15 (26.3)
Diplopia, n (%)	4 (12.5)	4 (7)
**The number of patients using csDMARDs, n (%)**	25 (55.6)	29 (40.3)
Azathioprine, n (%)	24 (53.3)	21 (29.2)
Methotrexate, n (%)	1 (2.2)	5 (6.9)
Sulfasalazine, n (%)	-	2 (2.8)
Interferon, n (%)	-	1 (1.4)
Cyclosporine, n (%)	1 (2.2)	5 (6.9)
**The number of patients using anti-TNFs, n (%)**	25 (55.6)	24 (33.3)
Infliximab, n (%)	-	2 (2.8)
Adalimumab, n (%)	25 (55.6)	19 (26.4)
Etanercept, n (%)	-	3 (4.2)
**Previous treatments, n (%)**		
Azathioprine	37 (84.1)	27 (37.5)
Methotrexate	2 (4.5)	9 (12.5)
Sulfasalazine	-	7 (9.7)
Interferon	8 (18.2)	1 (1.4)
Cyclosporine	9 (20.5)	7 (9.7)
Anti-TNFs	23 (51.1)	22 (30.6)
**The number of patients using glucocorticoids, n (%)**		
Oral	2 (4.4)	3 (4.2)
Topical	-	5 (6.9)

SD: Standard deviation; BMI: body mass index; csDMARDs: conventional synthetic disease-modifying antirheumatic drugs; n: number.

**Table 2:** Clinical features of the patients with common femoral vein wall measurements above 0.5 mm in the non-Behçet’s uveitis group.

Patients	Age (years)	Anatomical localization	R-CFV (mm)	L-CFV (mm)	OA	GU	PPL	EN	Venous thrombotic changes	Collaterals	Recanalization	Venous insufficiency	ISG	ICBD
1	44	intermediate	0.5	0.55	+	−	−	−	+	+	−	+	−	−
2	36	panuveitis	0.65	0.65	+	−	+	−	−	−	−	+	+	+
3	44	intermediate	0.55	0.6	+	−	−	−	+	−	−	−	−	−
4	42	intermediate	0.65	0.65	+	−	−	−	+	+	−	+	−	−
5	34	panuveitis	0.6	0.55	+	−	−	−	−	−	−	+	−	+
6	35	panuveitis	0.6	0.55	+	−	−	−	−	−	−	−	−	+
7	57	panuveitis	0.6	0.65	+	−	−	−	−	−	−	+	−	+
8	37	panuveitis	0.55	0.6	+	−	−	−	−	−	−	−	−	−
9	33	panuveitis	0.7	0.55	+	−	−	−	−	−	−	−	−	+
10	20	panuveitis	0.55	0.6	+	−	−	−	−	−	−	+	−	−

CFV: Common femoral vein; R: right; L: left; OA: oral aphthae; GU: genital ulcer; PPL: papulopustular lesions; EN: erythema nodosum; ISG: International Study Group Criteria; ICBD: International Criteria for Behçet’s Disease.
